# 2085: MyResearchHome@Duke—launch and adoption of a portal for the research community

**DOI:** 10.1017/cts.2017.56

**Published:** 2018-05-10

**Authors:** Rebecca Namenek Brouwer, Rebbecca Moen, Iain Sanderson, Ebony Boulware, Johanna O’Dell, Kellie Morris Browning

## Abstract

OBJECTIVES/SPECIFIC AIMS: Describe (1) the features of the first release of Duke’s myRESEARCHhome portal for researchers, and 2) the methods and results of adoption strategies METHODS/STUDY POPULATION: Through methods described previously (cite ACTS poster, 2016), the myRESEARCHhome portal team conducted a needs assessment to determine priorities for inclusion in the tool. Based on results of that assessment, the “minimal viable product” launched in June 2016 included the following features, organized into 9 distinct widgets: Access to all web-based research applications; ability to find and request research services; at-a-glance view of financial, protocol, and salary distribution information; access to financial and personnel reports; access to status of agreements and patents; access to CTSA-supported navigation services; visibility into required training and expiration dates; listing of announcements relevant to researchers; customized links area; ability to customize portal. The portal was developed using Ruby on Rails™, with a REACT grid framework. The development team, internal to Duke University, followed industry-standard best practices for development. After the initial release, the team employed several strategies to ensure awareness and adoption. Although written communications were an important factor for awareness, the presentations and hands-on studios proved most important. RESULTS/ANTICIPATED RESULTS: Use of the portal was directly related to in-person outreach efforts. There were small spikes after written communications, but strategies such as presentations, hands-on demonstrations, training sessions, and faculty meetings garnered the steadiest adoption rates. As of early January, 2017, almost 3000 users have interacted with the portal, with numbers rising steadily. There are an estimated 10,000+ faculty, staff, and trainees engaged in research at Duke. DISCUSSION/SIGNIFICANCE OF IMPACT: To maintain high adoption rates with the research community, engagement strategies must be ongoing. In addition to frequent in-person demonstrations, updates via written communications, and attendance at events, the portal team will employ a key adopt strategy—engaging the researchers in ongoing needs assessments. By maintaining the portal’s relevance to the needs of the research community, the tool can better improve the efficiency of research at a large academic medical center.

